# Performance of xenogeneic pulmonary visceral pleura as bioprosthetic heart valve cusps in swine

**DOI:** 10.3389/fcvm.2023.1213398

**Published:** 2023-08-02

**Authors:** Xiao Lu, Greg Kelley, Mengjun Wang, Xiaomei Guo, Ling Han, Ghassan S. Kassab

**Affiliations:** ^1^ Department of Bioengineering, California Medical Innovations Institute, San Diego, CA, United States; ^2^ Department of Research and Development, 3 DT Holdings, LLC, San Diego, CA, United States

**Keywords:** elastin and collagen, extracellular matrix, valve cusps, durability, pulmonary valvular replacement

## Abstract

**Objective:**

Bovine pericardium is common biological material for bioprosthetic heart valve. There remains a significant need, however, to improve bioprosthetic valves for longer-term outcomes. This study aims to evaluate the chronic performance of bovine pulmonary visceral pleura (PVP) as bioprosthetic valve cusps.

**Methods:**

The PVP was extracted from the bovine lung and fixed in 0.625% glutaraldehyde overnight at room temperature. The PVP valve cusps for the bioprosthetic valve were tailored using a laser cutter. Three leaflets were sewn onto a nitinol stent. Six PVP bioprosthetic valves were loaded into the test chamber of the heart valve tester to complete 100 million cycles. Six other PVP bioprosthetic valves were transcardially implanted to replace pulmonary artery valve of six pigs. Fluoroscopy and intracardiac echocardiography were used for *in vivo* assessments. Thrombosis, calcification, inflammation, and fibrosis were evaluated in the terminal study. Histologic analyses were used for evaluations of any degradation or calcification.

**Results:**

All PVP bioprosthetic valves completed 100 million cycles without significant damage or tears. In vivo assessments showed bioprosthetic valve cusps open and coaptation at four months post-implant. No calcification and thrombotic deposits, inflammation, and fibrosis were observed in the heart or pulmonary artery. The histologic analyses showed complete and compact elastin and collagen fibers in the PVP valve cusps. Calcification-specific stains showed no calcific deposit in the PVP valve cusps.

**Conclusions:**

The accelerated wear test demonstrates suitable mechanical strength of PVP cusps for heart valve. The swine model demonstrates that the PVP valve cusps are promising for valve replacement.

## Introduction

Transcatheter pulmonary valve replacement (TPVR) is becoming the treatment of choice in most congenital heart disease (CHD) patients with degeneration of prior right ventricular outflow tract repair. Right ventricular outflow tract (RVOT) dysfunction is a common hemodynamic challenge for children and adults with CHD, including patients with repaired tetralogy of Fallot (TOF), truncus arteriosus, and those who have undergone the Ross procedure for congenital aortic stenosis and the Rastelli repair for transposition of great vessels (1–4).

Recent advances in surgical techniques and perioperative care have dramatically improved the long-term outcome of CHD. Prior to the ground-breaking contribution of Dr. Bonhoeffer in the year 2000, open-heart surgery was the only modality to address RVOT dysfunction (5). The technical challenges of repeat redo cardiac surgery and the risk of myocardial injury related to repeated cardiopulmonary bypass adds to the complexity of the underlying CHD, which necessitated the search for an alternative approach. Since the life expectancy of these patients is improving, there is an increased demand for these procedures. Clinicians are now faced with a continuously growing population of adult patients with CHD, where most will require re-intervention in adulthood (6–8). Although transcatheter intervention to address RVOT obstruction, utilizing balloon angioplasty or stent implantation, provided relief of the RVOT obstruction, it came at the expense of pulmonary regurgitation with long-term detrimental effects (9). The introduction of TPVR serves as an alternative to address the stenosis and regurgitation in the same setting. Currently, pulmonary valve replacement is one of the most common procedures performed for adult CHD patients (10).

Cuspal calcification and degeneration, however, are major risks in pulmonary valve replacement (especially in younger patients) (11–13). Calcification of bioprosthetic heart valves in recipient patients causes deterioration of valvular function and eventually requires reoperation. Structural valve failure is caused by calcification which is histologically evident within three years of valve implantation (14). Mechanical stresses, including mounting the valve on various catheters and distortion of the valve or incomplete valve expansion, have been identified as risk factors for early valve failure. Calcification of bioprosthetic valves can be inhibited by reducing functional stresses through the modification of design and tissue properties (15). In this regard, our computational simulations showed that the mechanical stresses in elastic cusps are significantly smaller than those of more rigid cusps, where the PVP and bovine pericardium were used as the comparative cusps biomaterials (i.e., stress-strain relation of the two different materials were used), respectively (16). Hence, PVP cusps in bioprosthetic valve may mitigate calcification through stress reduction. Furthermore, our previous study demonstrated that the PVP is composed of abundant elastin fiber (17). It is well known that the elastin fibers are extensively covalent cross-linked (18), which enables PVP cusps to resist degradation and hence increase the longevity. Additionally, PVP vascular grafts/patches demonstrated very low thrombogenicity and low-inflammation in rodent and large animal (swine/canine) models (19–21). These observations formed the basis for the calcification mitigation hypothesis explored in the present study.

Here, we used glutaraldehyde-crosslinked PVP, to serve as the cusps of a bioprosthetic pulmonary valve in a swine model. The mechanical durability of the bioprosthetic valve was evaluated in accelerated fatigue testing. The bioprosthetic valves were implanted in the pulmonary outflow tract to replace a native pulmonary valve in the swine model for evaluation of the biocompatibility of the PVP bioprosthetic valve. The efficacy of the PVP bioprosthetic valve was postoperatively evaluated. The calcification, inflammation, and fibrosis of the PVP valve cusps were analyzed four months post-implant.

## Materials and methods

### Preparation of PVP valve cusps

The bovine lungs were obtained from Sierra for Medical Science (Whittier, CA 90607). The bovine PVP was separately extracted from the lungs with the aid of pressurized phosphate-buffered saline pumped into the interstitial space between the lung tissue and PVP. The PVP tissues were laid flat and rinsed with 4°C saline with 1% protease inhibitors (PMSF, phenylmethylsulfonyl fluoride) five times. The bovine PVP was fixed in 0.625% buffered glutaraldehyde (pH 7.4) overnight at room temperature to crosslink the proteins and diminish immune rejection. The PVP tissues were then stored in 0.25% buffered glutaraldehyde (pH 7.4) until valve construction. The thickness of PVP was measured using an electronic thickness gauge (Model 547-561S, Neoteck). Every piece of PVP tissue with dimensions of 9 (length) × 4 (width) cm was tailored for three valve cusps using a laser cutter. Three valve cusps with uniform thickness (±0.01 mm) were selected to assemble one bioprosthetic valve.

### Valve assembly and accelerated fatigue test

Three valve cusps were sewn onto the FoldaValve nitinol (self-expandable) stent (25 mm diameter) with a 6–0 suture (22). The Heart Valve Tester (Dynatek Labs M6 tester SN M6-102281) was used for the accelerated fatigue test of the PVP bioprosthetic valve (23). Six valves were loaded into the valve test chamber of the tester. The tester was filled with normal saline (0.90% w/v of NaCl) at 37⁰C. The tester was run with a system pressure of 120/80 mmHg and 800 cycles/min until 100 million cycles. The leaflet coaptation was observed for every valve. After completing 100M cycles, the valves were removed from the Heart Valve Tester. The valve cusps of each PVP bioprosthetic valve were examined visually under a microscope.

### Implant experiment

All animal experiments were performed in accordance with national and local ethical guidelines, including the Principles of Laboratory Animal Care, the Guide for the Care and Use of Laboratory Animals and the National Society for Medical Research, and an approved California Medical Innovations Institute IACUC protocol regarding the use of animals in research. Six domestic pigs (55 ± 5 kg) were used in the study. Animals were obtained from a certified vendor. The animals fasted for twelve hours before surgery. Appropriate aseptic techniques were followed for the survival surgery, including thorough scrubbing and wearing sterile garments. Intramural injections of TKX (4.4 mg/kg), consisting of a mixture of telazol (50 mg/ml), ketamine (25 mg/ml), and xylazine (25 mg/ml), were provided sedation. All animals were intubated and ventilated via a mechanical respirator with general anesthesia maintained via 1%–2% isoflurane and oxygen. The animals were monitored continuously for a surgical level of anesthesia. Joint tone, movement, blood pressure, and heart rate were all used to ensure a suitable surgical plane. Vital signs, including ECG, were monitored continuously throughout the procedures. A heating pad was used to maintain the body temperature of the animal. An intravenous (IV) line was placed percutaneously in the femoral vein to administer fluids and drugs. An isotonic saline drip was administered via a peripheral venous line (300 ml/h) to prevent dehydration. Heparin (∼100–200 IU/kg) was administered to achieve an activated clotting time of >200 s. Lidocaine (4 mg/kg), magnesium (20–50 mg/kg), and amiodarone (150 mg IV bolus) were administered to prevent arrhythmia deployment of the prosthetic valve.

The animal was placed in dorsal recumbency. The hair over the chest was clipped and cleaned. Baseline intracardiac echocardiography (ICE) measurement was performed. The animal was covered with sterile surgical drapes. Baseline angiography and cardiac pressure measurements were performed. The chest was opened through a midline sternotomy. As for heart exposure, an appropriate size of the sheath was placed from the right ventricular apex. The delivery system (transventricular catheter) for the bioprosthetic valve was advanced over a guidewire positioned in the pulmonary artery and positioned for deployment at the junction of the RVOT and pulmonary artery. The bioprosthetic valve was carefully deployed over the native pulmonary valve at the outflow tract. The incision at the right ventricular apex was closed by suture continuously. The sternum was closed with four or five stainless steel sutures. The muscle layer and subcutaneous tissue was closed with an absorbable suture, while the negative chest pressure was restored through a chest tube. The skin was closed with surgical staples. Fluoroscopy was used to evaluate whether the PVP bioprosthetic valves migrated from the RVOT and pulmonary artery junction. ICE was used to assess the open and coaptation of the PVP bioprosthetic valves. Clopidogrel (75 mg/day) and Aspirin (325 mg/day) were administered orally for survival durations as the general postoperative treatment of vascular surgery to prevent blood clots.

On the terminal day, the six animals were anesthetized and heparinized. Fluoroscopy and ICE were performed to assess the position and function of the PVP bioprosthetic valves. The animals were euthanized. The chest was re-opened to expose the heart. Visual assessment of fibrosis or inflammation was completed. The animal was euthanized. The heart was excised for visual assessment of the PVP bioprosthetic valve in the pulmonary artery outflow tract. The PVP bioprosthetic valve was amputated from the adjacent aortic wall for examination with the aid of stereomicroscope. After fixation with 4% paraformaldehyde, the cusps with the entire tissue complex were carefully dissected from the stent. A segment on circumferential plane and a segment on axial plane in each cusp were sliced for histologic analyses.

### Histological analyses

The segments were dehydrated through graded ethanol and embedded in paraffin. The segments were sectioned 3 *μ*m thick for histological staining. After deparaffinization and rehydration, the routine processing was performed for hematoxylin-eosin (HE) staining for overall morphologic features, Masson's trichrome staining for collagen, von Kossa staining (Calcium Stain Kit, NC1969831, Fisher Scientific) for calcium phosphates, and Alizarin Red staining (Millipore Sigma) for calcium deposition. In addition, Oil Red O Staining (Lipid Stain Kit: ES4814, Azer Scientific) and Perls Prussian Blue staining (Iron Stain Kit: ES3402, Azer Scientific) were used to evaluate lipid and iron (intravalvular hemorrhage) depositions. Each section was analyzed using light microscopy. We also used immunofluorescence to visualize elastin and collagen fibers in the PVP cusps of bioprosthetic valves. Besides the routine processing for deparaffinization and rehydration, the processing for immunofluorescence were performed, i.e., antigens retrieval, blockage in 3% bovine serum albumin, rinse with PBS, etc. The tissue sections were incubated with primary antibodies, anti-elastin (Cat #:sc17480, 1/30 dilution with PBS contained 0.25% Triton and 2.5% donkey serum, Santa Cruz Biotechnology), anti-collagen (Cat.#:ab7778, 1/25 dilution with PBS contained 0.25% Triton and 2.5% donkey serum, & ab6586, 1/20 dilution with PBS contained 0.25% Triton and 2.5% donkey serum, Abcam), MMP-9 (Cat.#:sc21733, 1/50 dilution, Santa Cruz Biotechnology), and fibrin (Cat.#:350, 1/20, American Diagnostic Inc), respectively. After PBS rinse, the sections were incubated with fluorescent dye-conjugated secondary antibodies (Cat. #A10040, A10036, A11081, & A11058, 1/100, dilution with PBS contained 0.25% Triton and 2.5% donkey, serum, Thermo Fisher Scientific). Fluorescent wheat germ agglutinin (Cat.#:w11261, 1/100 dilution, Thermo Fisher Scientific) was used to label glycosaminoglycans and proteoglycans in extracellular matrix (ECM) (24). The images were obtained using a fluorescence microscope (Eclips TS2R FL, Nikon).

### Statistics

Average and standard deviation are reported for the various measurement parameters.

## Results

**Figure 1:**
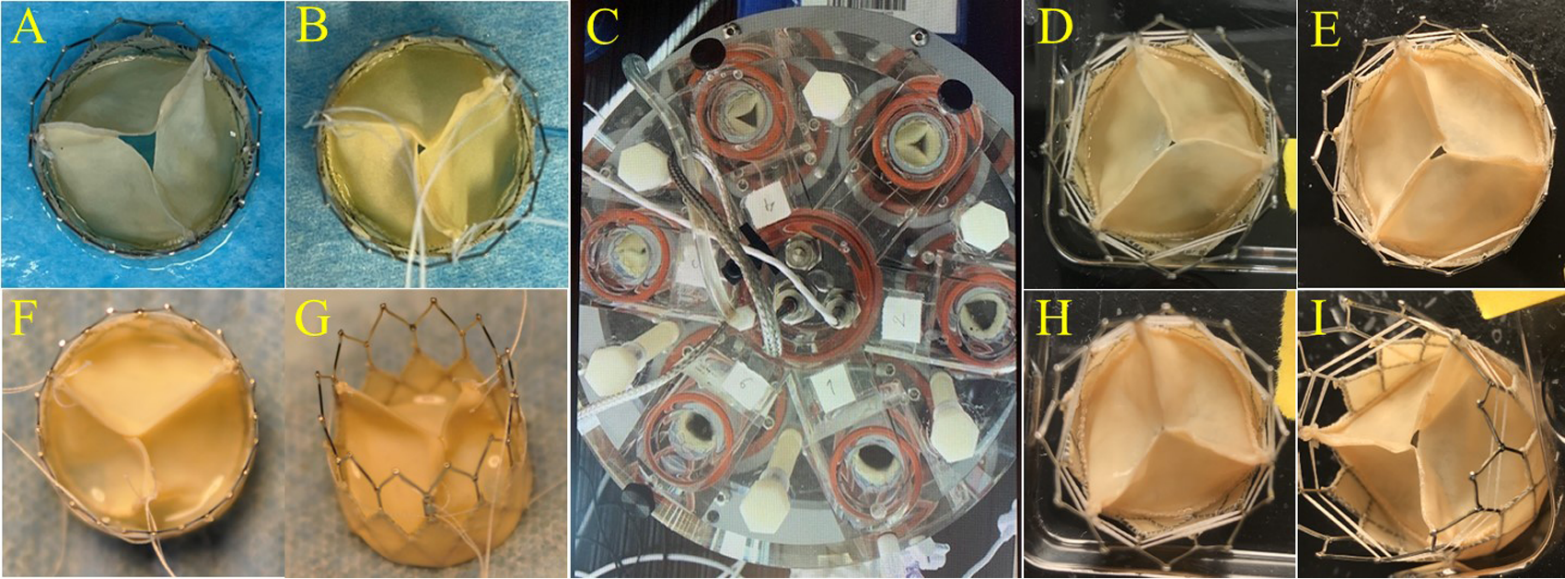
PVP cusps in bioprosthetic valve and accelerated wear test. Examples of diverse thicknesses of PVP cusps in bioprosthetic valves. (**A,B,F,G**) Representation of the PVP bioprosthetic valves before testing in accelerated wear/fatigue tester. (**A**) 0.17 mm, (**B**) 0.22 mm, (**F**) 0.25 mm, and (**G**) 0.26 mm. (**C**) Representation of accelerated wear/fatigue test in Dynatek Labs M6 tester. The medium was saline. Parameters were 800 cycles/min, 120/80 mmHg for systolic and diastolic pressures, and 37°C of testing temperature. The cycles reached 100 million cycles while the tester ran for four months. (**D**,**E**,**H**,**I**) Representation of the PVP bioprosthetic valves after testing for 100 million cycles. No significant tear/damage was observed on the PVP cusps.

We harvested approximately 400 cm^2^ PVP with a uniform thickness from each lung set. The thickness of PVP varied due to the different age/weight of animals and regions at the lung surface. Generally, the thickness of bovine PVP ranged from 110 to 280 μm. Although it is thinner than bovine pericardium, the PVP can be handled and sewn for leaflets and skirts of bioprosthetic valves. Four examples of bioprosthetic valves with diverse valve cusps thicknesses (0.17 to 0.26 mm) are presented in Figures 1A–B and 1F–G. All three valve cusps in one PVP bioprosthetic valve are symmetric with coaptation at ∼3 cm hydraulic pressure. Six PVP bioprosthetic valves with valve cusp thicknesses of 0.17 ± 0.01 mm (*n *= 2), 0.22 ± 0.015 mm (*n *= 2), and 0.25 ± 0.017 mm (*n *= 2), respectively, were collected for accelerated wear/fatigue tests. The prosthetic valves were mounted in the 6-chambers in the Dynatek Labs M6 tester, respectively. In the Heart Valve Tester, normal valve cusps coaptation was observed. All six PVP bioprosthetic valves completed 100 million cycles. All valve cusps had opened and had coaptation at the end of 100 million cycles (Figure 1C). No significant tears were observed for the valve cusps of each PVP bioprosthetic valve that were examined visually under a microscope (Figures 1D–E and 1H–I).

**Figure 2:**
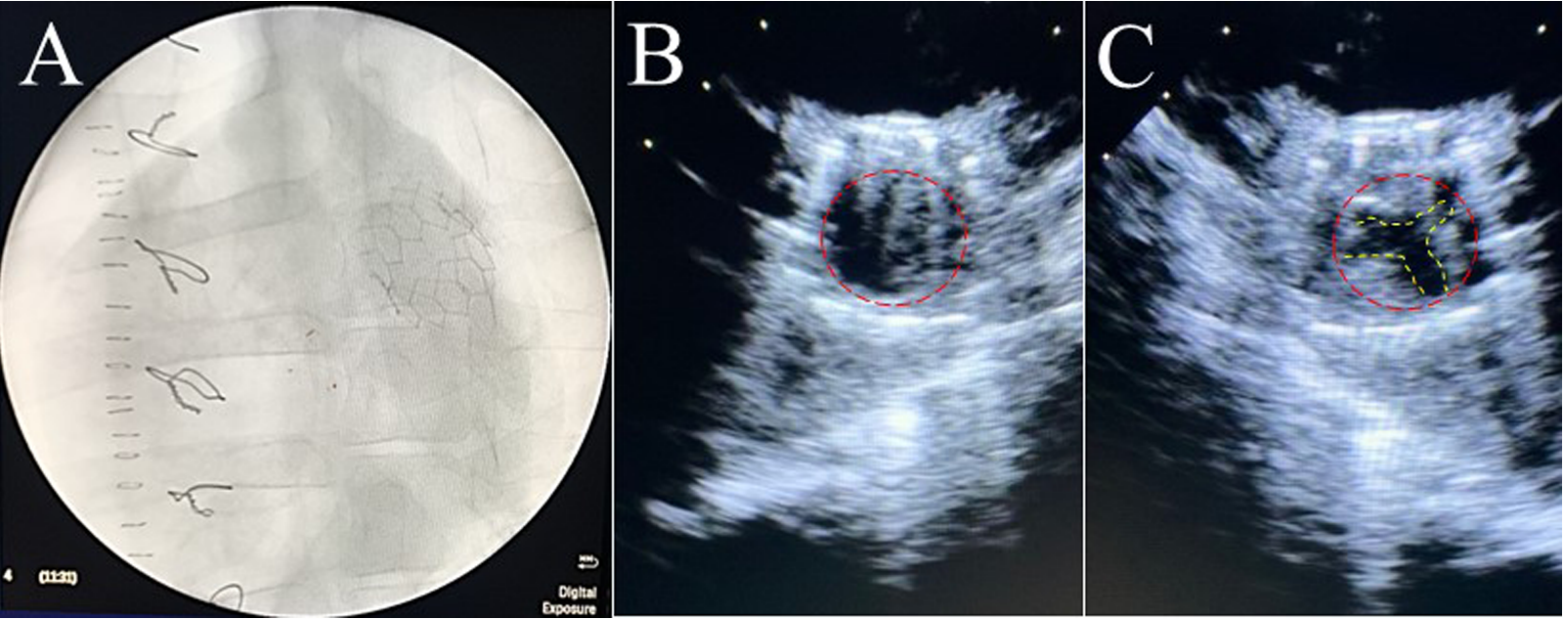
Fluoroscopic and ultrasound (ICE) images of a bioprosthetic valve in the pulmonary artery outflow tract of a pig. (**A**) Fluoroscopic image visualized the stent of a bioprosthetic valve in the pulmonary artery outflow tract of a pig. (**B**) Ultrasound image (transverse plane) showed the opening of a bioprosthetic valve in the pulmonary artery outflow tract of a pig. (**C**) Ultrasound image (transverse plane) showed the coaptation of a bioprosthetic valve in the pulmonary artery outflow tract of the pig.

**Figure 3:**
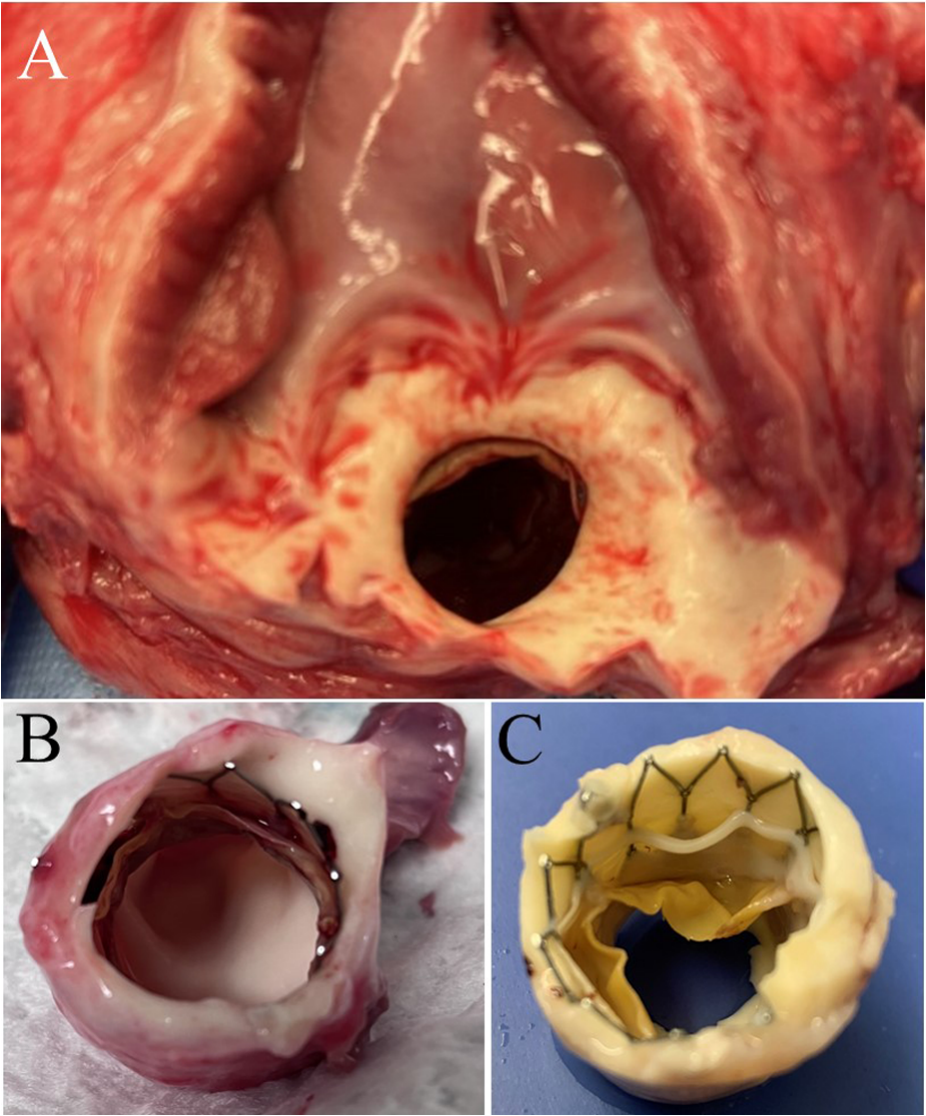
Postmortem images of bioprosthetic valves implanted in swine pulmonary artery for four months. (**A**) A bioprosthetic valve in the pulmonary artery of a pig. No observation of fibrosis and inflammation in the pulmonary artery outflow tract and myocardium. (**B**) The excision of extrapulmonary arterial tissues and myocardium further exposed the bioprosthetic valve in the pulmonary artery. (**C**) The bioprosthetic valve in the pulmonary artery was fixed in 4% paraformaldehyde for twenty-four hours.

Six PVP bioprosthetic valves with cusps thicknesses of 0.17 ± 0.01 mm (*n *= 3) and 0.22 ± 0.015 mm (*n *= 3) were implanted in six pigs, respectively. While the PVP bioprosthetic valves were implanted at the junction of RVOT and pulmonary artery in pigs, no complications were observed in any animal during the postoperative period. In the terminal study, we did not observe any migration of the PVP bioprosthetic valves in the junction of RVOT and pulmonary artery until postoperative four months (Figure 2A). No right ventricular dilation was observed in fluoroscopy. The valve cusps opening and coaptation were observed by ICE (Figures 2B,C). In the post-mortem examination, we did not observe any thrombotic deposit, inflammation, or fibrosis in the heart and pulmonary artery (Figure 3A). Further dissection to expose the PVP bioprosthetic valves showed no thrombotic deposit, inflammation, or fibrosis on the valve cusps and skirt (Figure 3B). When the PVP bioprosthetic valves were isolated, we did not observe any calcific deposit on the valve cusps, valve skirt, or aortic wall (Figure 3C).

**Figure 4:**
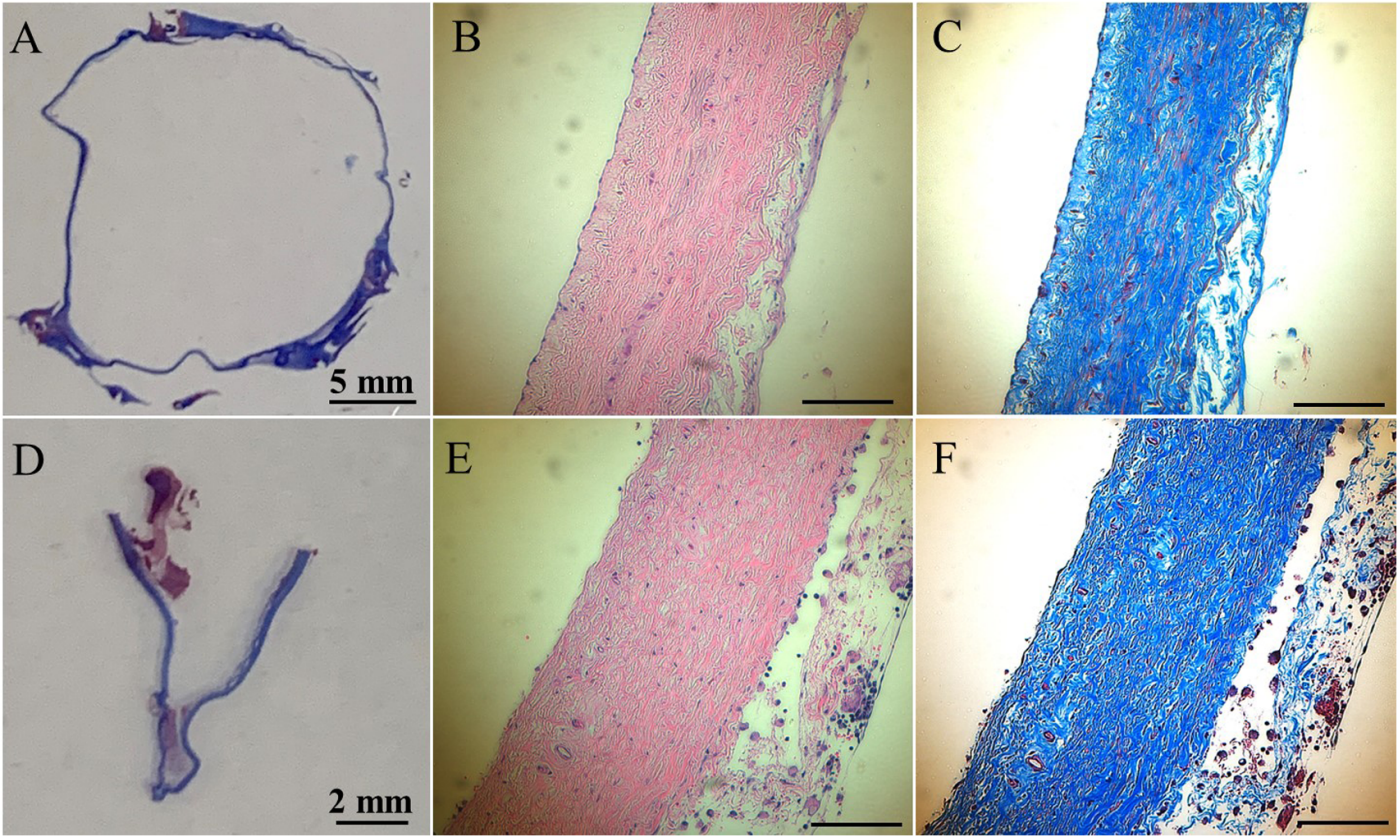
HE and trichrome stains of the PVP bioprosthetic valves. (**A**–**C**) Represent the transversal plane of the PVP cusp in bioprosthetic valve implanted in the pulmonary artery outflow tract for four months. (**D**,**E**,**F**) Represent the longitudinal plane of the PVP cusp in bioprosthetic valve implanted in the pulmonary artery outflow tract for four months. (**A**,**D**) Are macroscopic pictures of trichrome stains. (**B**,**E**) Are microscopic pictures of HE stain. Bars: 0.1 mm. (**C**,**F**) Are microscopic pictures of trichrome stain. Bars: 0.1 mm.

**Figure 5:**
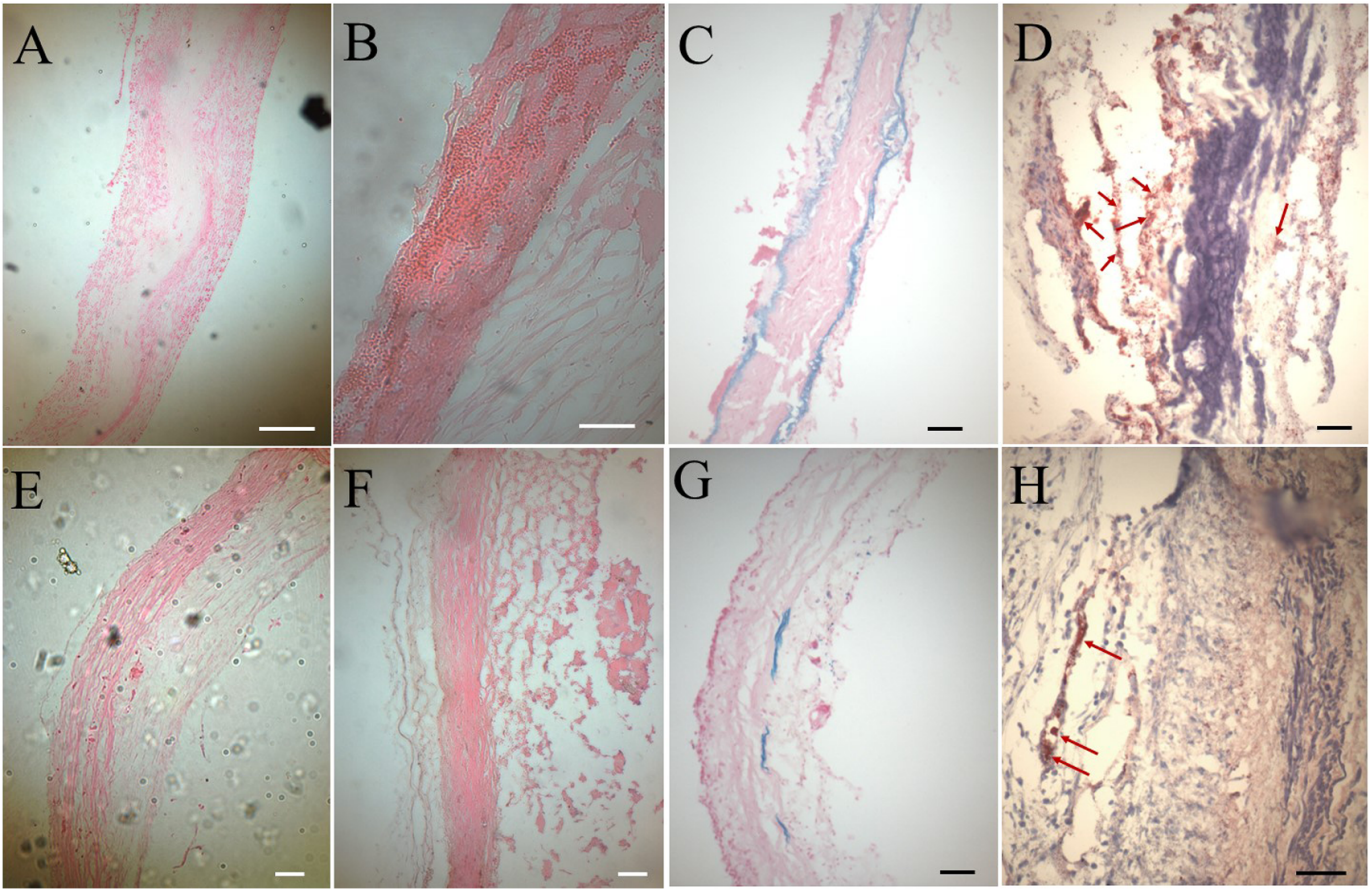
Histological analyses on calcification, iron deposit, and lipid deposit. (**A**–**D**) Represent the transversal plane of the PVP cusp in bioprosthetic valve implanted in the pulmonary artery outflow tract for four months. (**E**–**H**) Represent the longitudinal plane of the PVP cusp in bioprosthetic valve implanted in the pulmonary artery outflow tract for four months. (**A**,**E**) Are microscopic pictures of the von Kossa stain. (**B**,**F**) Are microscopic pictures of the Alizarin Red stain. (**C**,**G**) Are microscopic pictures of the iron stain. Pink: nuclei. Dark blue: iron deposit. (**D**,**H**) Are microscopic pictures of the Oil Red O stain (lipid deposit). Dark violet: nuclei. Red: lipid deposit. Red arrows: pointing lipid deposit. White and black bars for **A** to **H**: 0.1 mm.

**Figure 6:**
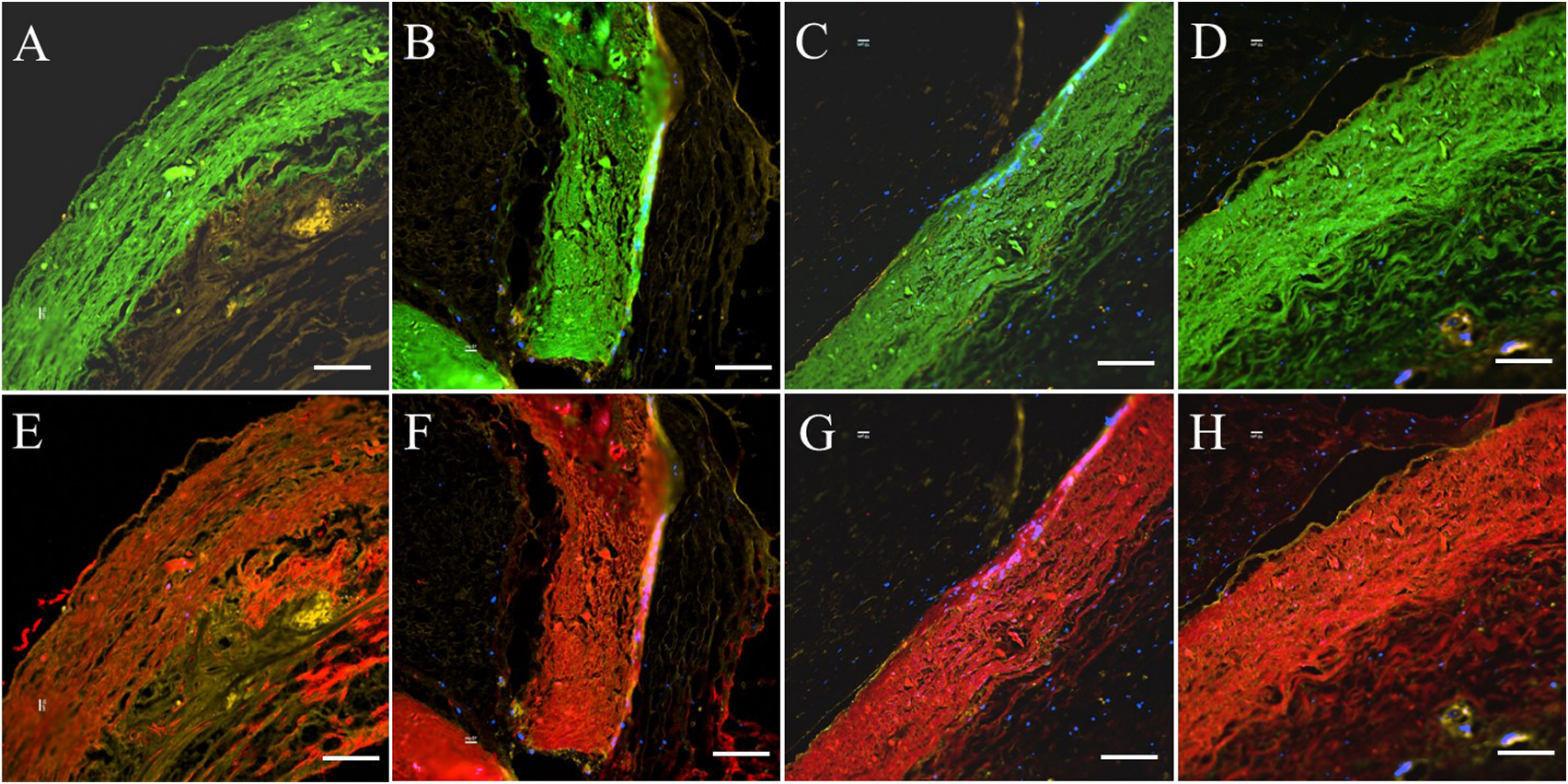
Elastin and collagen fibers of PVP cusp in the bioprosthetic valves implanted in pigs for four months. Four areas of interest are randomly selected for representation. (**A**–**D**) Represent elastin (green) fibers in the PVP cusp. (**E**–**H**) Represent collagen (red) fibers in the PVP valve cusps. (**A**,**B**,**E**,**F**) Represent the transverse plane of the PVP valve cusps. (**C**,**D**,**G**,**H**) represent the longitudinal plane of the PVP valve cusps. Bars for **A** to **H**: 100 μm.

**Figure 7:**
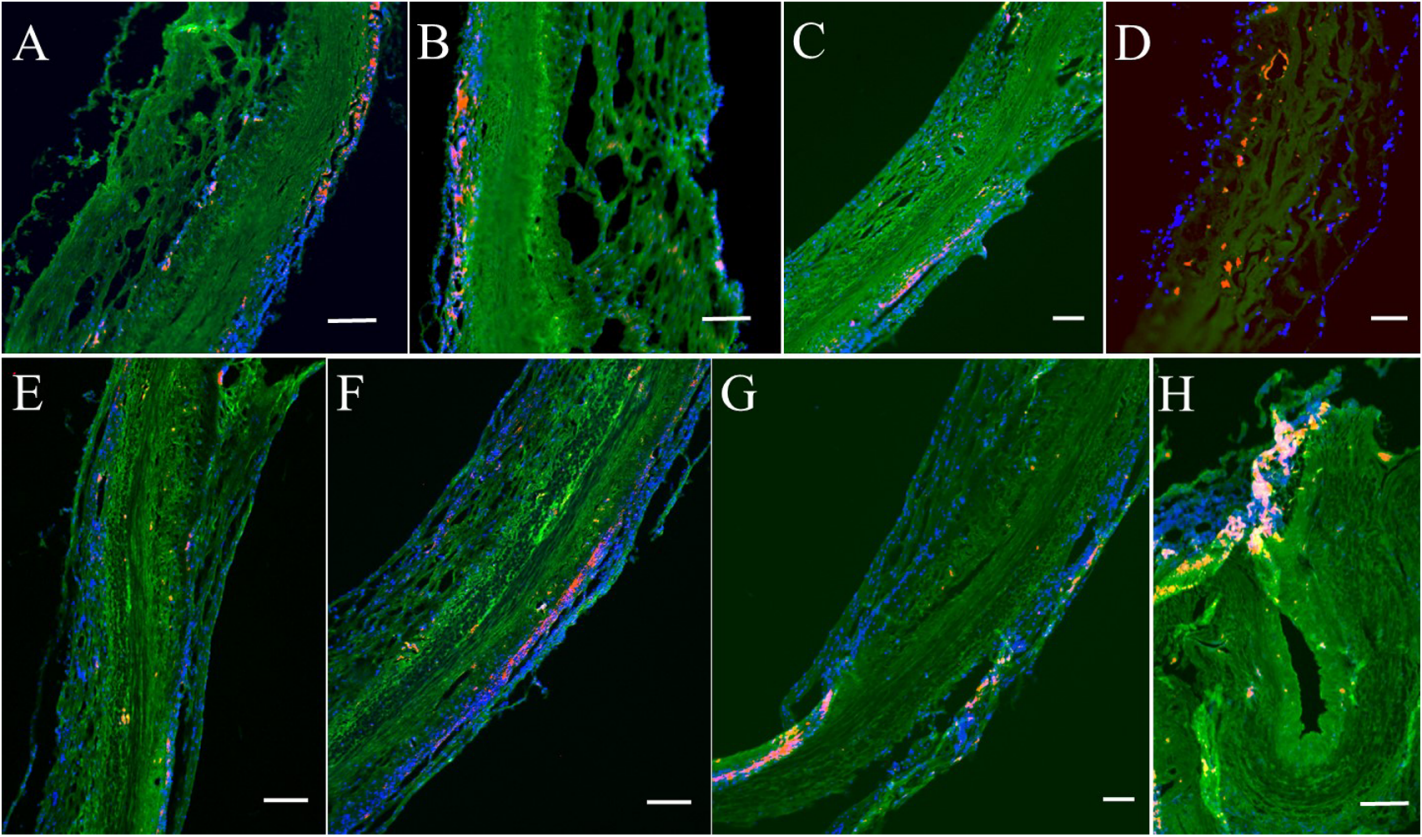
Immunofluorescence analyses on MMP9 and fibrin for post-implanted four months. (**A**–**D**) Represent MMP9 expression. Red/Orange: MMP9. Green: Fluorescent wheat germ agglutinin (WGA). Blue: Nuclei. (**E**–**H**) Represent fibrin expression. Red/Orange: Fibrin. Green: Fluorescent wheat germ agglutinin (WGA). Blue: Nuclei. Bars for **A** to **H**: 100 μm.

In histologic analyses, all tissue slides were carefully reviewed in accordance with a pathologist's instruction. The structure of valve cusps remained intact and functional. Some examples are shown in Figures 4A–F. The valve cusps of PVP bioprosthetic valves did not show thickening for the four-month duration (Figures 4A–F). We did not observe any tearing or degradation in the valve cusps of PVP bioprosthetic valves (Figures 4B,C,E,F). The collagen fibers (blue) in the valve cusps were well integrated for the four-month duration (Figures 4C,F). A few host cell migrations (Red) were observed within the valve cusps (Figures 4C,F). The calcific deposit should be represented black/dark grey in the von Kossa stain (Figures 5A,E) or dark red in the Alizarin Red stain (Figures 5B,F). We did not observe any black spot or dark red plaque in the valve cusps (Figures 5A,B,E,F) for the four-month duration. The iron deposit was observed in 1 of 6 PVP bioprosthetic valvular implants (Figures 5C,G). The iron deposit was mainly observed in the external region of the PVP cusps. The lipid deposit was observed in 1 of 6 PVP bioprosthetic valvular implants (Figures 5D,H). In the one implant where lipid deposition was observed, it was only seen in the external region of the PVP cusps. Using immunofluorescence microscopy, we examined the integration and compaction of elastin and collagen fibers in the valve cusps of PVP bioprosthetic valves. The elastin fibers in the valve cusps remained intact and robust during the four-month period (Figures 6A–D). The collagen fibers in the valve cusps were also compact and continuous for the four-month duration (Figures 6E–H). The MMP9 expression was observed in 3 of 6 PVP bioprosthetic valvular implants (Figures 7A–D). The MMP9 expression was observed in external regions of PVP cusps in 2 bioprosthetic valvular implant (Figures 7A–C). In one bioprosthetic valvular implant, the MMP9 expression was observed in internal regions of PVP cusps (Figure 7D). The fibrin expression was observed in 1 of 6 PVP bioprosthetic valvular implants (Figures 7E–H). The fibrin expression was mainly in external region of the PVP cusps (Figures 7E–G) and the commissure of cusps (Figure 7H).

## Discussion

This is the first study using bovine PVP valve cusps as bioprosthetic heart valves in large animal models. The implanted PVP bioprosthetic valves remained at the junction of RVOT and pulmonary artery of pigs without migration for the 4-month period. There were no signs of calcification and degradation in the PVP pulmonary bioprosthetic valve. No thrombotic deposit, fibrosis, or inflammation were observed in PVP valve cusps of bioprosthetic valves in gross examination or histologic analyses. Histologic and immunofluorescence microscopic analyses did not reveal any collagen and elastin fibers degradation in the PVP valve cusps.

Hemodynamically significant RVOT dysfunction (regurgitation due to valvular dysfunction) is commonly encountered in adulthood in patients who have undergone previous surgical repair for several conditions, including TOF, pulmonary atresia with ventricular septal defect, congenital pulmonary stenosis, truncus arteriosus, previous Ross procedure for congenital aortic stenosis, and Rastelli repair for transposition of great vessels. Pulmonary valve replacement has become one of the most common procedures for pediatric and CHD patients (4). Surgical pulmonary valvular replacement (SPVR) still remains the gold standard for patients with congenital heart diseases (25). TPVR can be a reliable and safe alternative to SPVR in patients that have undergone prior surgeries for congenital heart disease (25). Compared to SPVR, TPVR was associated with a significant reduction in risk for all-cause mortality at the longest available follow-up, recurrent pulmonary regurgitation, and thirty-day hospitalization, while the risk for post-procedural infective endocarditis was significantly higher (25). Improvements for TVPR include features such as a lower introducer profile (currently, delivery systems are 16–24 Fr size), low inflammatory response, no infection, long durability, low opening resistance with maximal valve area, fast and reliable closure, and non-thrombogenicity.

The thickness of bovine PVP ranges from 110 to 280 μm which is significantly smaller than that of the bovine pericardium (>200 μm). It is known that approximately 40% of the bulk size of the trans-catheter valve stems from the valve cusps; i.e., the delivery system can be reduced accordingly when the tissue of valve cusps is thinner. Therefore, the PVP can substantially reduce the profile of the delivery system. Thinner PVP valve cusps would also reduce the tissue's degree of crimping, which may cause damage and hence potential failure (tearing and calcification). Although it is thinner than bovine pericardium, we demonstrate in the study that the PVP valve cusps have no tear or degradation after 100 M cycles in an accelerated fatigue/wear test. We have demonstrated in the previous investigation that the PVP graft has similar burst pressure to the artery (19). Therefore, the mechanical strength of the PVP is suitable for the valve cusps of a bioprosthetic heart valve. Furthermore, we have also demonstrated in previous studies excellent biocompatibility and non-thrombogenicity of PVP vascular graft and patch in animal models (19–21). The present study underscores the excellent biocompatibility and non-thrombogenic property of the PVP bioprosthetic pulmonary valve in a large animal model.

The PVP bioprosthetic valve exhibited excellent resistance to calcification and inflammation in the study, which is consistent with our previous studies (19–21). This suggests the potential for improved long-term durability than the current bioprosthetic valves using bovine pericardium. It is known that the collagen and elastin debris in valvular prosthesis due to degradation can induce calcification. The high mechanical stress in the valvular prosthesis is one of the causes of the degradation of collagen and elastin fibers. Our previous studies show that the PVP contains abundant elastin, and the ratio of elastin to collagen is about 1:1 (17). In contrast, the pericardium and peritoneum have a collagen to elastin ratio > 40.0:1 (26). Elastin and collagen are the major extracellular matrix proteins (27–29). Elastin is a potent autocrine regulator of vascular smooth muscle cell activity and inducer of actin stress fiber organization. Elastin also regulates myofibroblasts activity and promotes quiescent fibroblasts (convert from genotype to phenotype state) (30–33), which may balance the proliferation on the PVP valve cusps. Elastin largely retains its elasticity after chemical/physical treatments to mitigate immune rejection (34). Our simulation shows that the elasticity due to elastin may reduce the stress in the PVP valve cusps of the bioprosthetic valve in the heartbeat cycle (16). In the histologic analyses of postmortem, collagen and elastin fibers were intact in the PVP valve cusps of the bioprosthetic valve in a large animal study for four months (Figures 4, 6). This correlates with the lower stresses induced in the leaflets due to the higher elasticity of the PVP. Furthermore, the minor MMP-9 expression in the adjacent tissue of the PVP cusps (Figures 7A–D) also supports that there was little degradation of collagen and elastin in the PVP cusps. It is known that lipid deposit is a risk factor of degradation for bioprosthetic valves (35, 36). The histological analysis showed a minor lipid deposit in adjacent tissue of the PVP cusps (Figures 5D,H), which suggests that lipid induced enzymatic precipitation and degradation are not implicated in the calcification and degradation of PVP cusps.

Bioprosthetic valve thrombosis (BPVT) is a major cause of bioprosthetic valve degeneration and often has an elusive presentation causing delayed recognition and treatment (37). BPVT is a recognized complication of prosthetic aortic valves and can be found in up to 13% of patients after transcatheter implantation (38). BPVT may result in valve dysfunction, possibly related to degeneration and recurrence of patient symptoms, or remain subclinical (34). Recent reports have suggested a high incidence of subclinical cusps thrombosis following bioprosthetic aortic valve replacement (25, 39, 40). In previous studies, we demonstrated the non-thrombogenicity of the PVP as a vascular graft and patch of artery and vein (20, 21). In histological analyses, iron deposit, product of hemoglobin degradation, was found in only one of six PVP bioprosthetic valves, and the iron deposit was located at the boundary between the PVP cusps and adjacent tissue (Figures 5C,G), which suggests a minor thrombosis at the surface of the PVP cusps. Minor fibrin expression in the adjacent tissue of the PVP cusps (Figures 7E,F) also indicates that there was no intravalvular hemorrhage. The thrombosis resistance of the PVP valve cusps of the bioprosthetic valve is verified in a large animal model. Therefore, the non-thrombogenic PVP valve cusps may mitigate the complications of BPVT to enhance the longevity of the bioprosthetic valve. The longevity of bioprosthetic heart valve, however, is a major hurdle in the clinic. Calcification and degeneration significantly decrease the longevity of bioprosthetic valves especially in younger patients. The major hurdle that remains is translation of our current animal studies to patients where significant co-morbidities in patients may play a role in the outcome, i.e., no animal model truly recapitulates the human conditions.

## Study limitations

In this study, we did not include a control group of bioprosthetic valves as the cost of the valves was beyond our budget. We refer to historical observations in the literature on pericardium cusps in bioprosthetic heat valve, which show significant calcification in the glutaraldehyde-fixed pericardial cusps at 3-month implants in sheep/pig model (41, 42). The post-implantation for 4 months in this study that shows no calcification which is a major milestone. Despite the lack of control, experimental and clinical literature have clearly demonstrated the propensity to calcification and tissue failure under fixation which is the standard of care clinically to eliminate the immune response. Therefore, despite the lack of control group, our finding of no-calcification of glutaraldehyde fixed tissue in a 4-month duration is very significant and warrants future clinical investigations.

Glutaraldehyde-fixation is one of risk factors of calcification in bioprosthetic heart valve (35, 36). Various processes for biological tissue, such as decellularization, different crosslink agents, and tissue engineering technology, have been developed to mitigate immune rejection, inflammation, fibrosis, etc (43, 44). To compare with the literature on pericardium cusps in bioprosthetic valve, glutaraldehyde fixation was used in this study. The updated technology for processing PVP biomaterial will be investigated in future.

Although the PVP bioprosthetic valves were delivered into the junction of RVOT and pulmonary artery using a catheter system, we did not achieve transfemoral vein delivery. The thinner cusps do reduce the profile of the catheter in the delivery system, however, which provides the opportunity to develop a 12 Fr catheter in the delivery system using a transfemoral vein. The observations in this study (e.g., no thrombotic deposit, no inflammation, no calcific deposit, etc.) are still applicable regardless of the delivery route.

## Clinical perspectives

The PVP valve cusps satisfy the basic mechanical strength requirements for a bioprosthetic heart valve, despite being thinner than the bovine pericardium. The implantation of PVP bioprosthetic valves in RVOT demonstrates PVP valve cusps' resistance to thrombotic deposits, inflammation, fibrosis, and calcification. Therefore, the PVP tissue is a very promising biological material to serve as the valve cusps of bioprosthetic valves for heart valvular replacement.

## Data Availability

The original contributions presented in the study are included in the article/Supplementary Material, further inquiries can be directed to the corresponding author.
